# Predicting clinical response to everolimus in ER+ breast cancers using machine-learning

**DOI:** 10.3389/fmolb.2022.981962

**Published:** 2022-10-11

**Authors:** Aritro Nath, Patrick A. Cosgrove, Jeffrey T. Chang, Andrea H. Bild

**Affiliations:** ^1^ City of Hope Comprehensive Cancer Center, Department of Medical Oncology and Therapeutics, Monrovia, CA, United States; ^2^ Department of Integrative Biology and Pharmacology, University of Texas Health Science Center at Houston, Houston, TX, United States

**Keywords:** machine-learning, biomarker, everolimus, estrogen receptor positive breast cancer, prognostic model, random forest, feature selection

## Abstract

Endocrine therapy remains the primary treatment choice for ER+ breast cancers. However, most advanced ER+ breast cancers ultimately develop resistance to endocrine. This acquired resistance to endocrine therapy is often driven by the activation of the PI3K/AKT/mTOR signaling pathway. Everolimus, a drug that targets and inhibits the mTOR complex has been shown to improve clinical outcomes in metastatic ER+ breast cancers. However, there are no biomarkers currently available to guide the use of everolimus in the clinic for progressive patients, where multiple therapeutic options are available. Here, we utilized gene expression signatures from 9 ER+ breast cancer cell lines and 23 patients treated with everolimus to develop and validate an integrative machine learning biomarker of mTOR inhibitor response. Our results show that the machine learning biomarker can successfully distinguish responders from non-responders and can be applied to identify patients that will most likely benefit from everolimus treatment.

## Introduction

Breast cancer is now the most commonly diagnosed malignancy and cause of cancer-related death in women worldwide (Houghton and Hankinson, 2021). In the United States, one in eight women will be diagnosed with breast cancer throughout their lifetime (Siegel et al., 2019). At the molecular level, nearly 3 in 4 breast cancers display increased expression of the estrogen receptor (ER+) and do not express the human epidermal growth factor receptor 2 (HER2-). The primary systemic treatment of ER+/HER2- breast cancer is endocrine therapy, which targets the dependency of these tumors on the estrogen signaling pathway for proliferation. These include selective estrogen receptor modulators (SERMs) like tamoxifen, selective estrogen receptor degraders (SERDs) like fulvestrant, and aromatase inhibitors (AIs) like exemestane (Smith and Dowsett, 2003; Patel and Bihani, 2018).

Patients with primary or early-stage ER+/HER2- breast cancer generally have a favorable outlook, with excellent 5-year survival rates on endocrine therapy, even without the use of adjuvant chemotherapy (Early Breast Cancer Trialists' Collaborative et al., 2012). However, the response rates tend to be lower in patients with metastatic disease, with only 30% of the patients displaying tumor regression on endocrine therapy (Osborne and Schiff, 2011). This outcome has been attributed to primary or acquired endocrine resistance in progressive tumors. Studies have shown that advanced and metastatic ER+ breast cancers can develop endocrine resistance through various mechanisms, such as mutations in the ER-alpha gene, amplification/overexpression of epidermal growth factor and fibroblast growth factor receptor family genes, and activation of downstream signaling *via* the PI3K/AKT/mTOR signaling pathway (Musgrove and Sutherland, 2009; Clarke et al., 2015). Consequently, add-on drugs that target the resistance mechanisms, such as the PI3K inhibitor alpelisib and the mTOR inhibitor everolimus, have shown promising results in clinical trials for advanced ER+ breast cancers. For example, the SOLAR-1 trial reported an improvement of median overall survival in *PIK3CA* mutated cancers from 5.7 months in the fulvestrant group to 11 months in the alpelisib plus fulvestrant group. The BOLERO-2 trial showed significant improvement in progression-free survival in post-menopausal ER+ breast cancers from 2.8 months on exemestane alone to 6.9 months on everolimus plus exemestane (Baselga et al., 2012).

Unlike the PI3K inhibitors, currently, there are no clinically relevant biomarkers available for the selection of everolimus as the treatment for ER+ breast cancers. In the absence of suitable guidelines, this choice is primarily based on patient and caregiver preferences. We have previously shown that effective prognostic and response biomarkers can be developed from the baseline (pre-treatment) transcriptomes of the tumors using systems biology and machine learning (Nath et al., 2019; Nath et al., 2022). In this study, we apply a machine learning framework to develop a novel biomarker model to predict clinical response to everolimus. We adopt a hybrid approach that integrates signatures of treatment response from well-controlled *in vitro* experimentation of cell lines treated with everolimus with empirical signatures derived from the baseline tumor transcriptomes of 23 patients. Using this approach, we develop and validate a predictive model of everolimus response and demonstrate its potential application in identifying candidates for mTOR inhibitor treatment.

## Materials and methods

### Breast cancer cell line culture and drug treatment

Nine ER+/HER2- breast cancer cell lines were used in this study. CAMA-1, LY2, and MCF7 cell lines were grown and cultured in Dulbecco’s Modified Eagle Medium (DMEM, Gibco, Cat# 11995073) + 10% heat-inactivated Fetal Bovine Serum (FBS, Sigma-Aldrich, Cat # F4135) + 1x antibiotic-antimycotic (Gibco, Cat# 15240062). T47D, BT-483, ZR-75-1, HCC1428, MDA-MB-134-VI, and MDA-MB-175-VII were grown and cultured in RPMI-1640 (Gibco, Cat# 11875119) + 10% heat-inactivated FBS + 1x antibiotic-antimycotic. Cell lines were authenticated by STR profiling (at *City of Hope Integrative Genomics Core*) and tested negative for mycoplasma contamination using MycoAlert Mycoplasma Detection Kit (Lonza, Cat# LT07-118).

To determine an effective concentration of everolimus and exemestane for everolimus plus exemestane signature each cell line was plated at 1,000 cell/well in a 384-well flat bottom TC-treated plate (Corning, Cat# 3764) and allowed to adhere at 37°C humidified incubator + 5% CO_2_. After 24hrs post-plating, cells were incubated with a dose-response of everolimus or exemestane or 0.2% DMSO control for 4 days (40 μL total volume). Viability was assessed as a measure of total ATP using the CellTiter-Glo assay (Promega, Cat# G7573) according to manufacturer instructions. See Supplementary Table S1 and Supplementary Figure S1.

Each cell line was plated at 250,000 cells/well in 2 ml of the respective culture media on a 6-well tissue culture treated plate (Costar, Cat# 3506) and allowed to adhere at 37°C humidified incubator + 5% CO_2_. After 24 h post-plating, the cells were treated with either 0.2% DMSO (control) or a combination of 0.5 nM everolimus + 25 μM exemestane (Selleck Chemicals, Cat# S1120, S1196 respectively) in their respective culture media. Following treatment, the cells were incubated for 6 h at 37°C + 5% CO_2_ in a humidified incubator.

### Cell lines RNA extraction, cDNA synthesis, library preparation, and sequencing

After 6 h of treatment, the plated cells were rinsed one time with ice-cold 1x PBS (Gibco, Cat# 10010049) followed by collection *via* cell scrapping in ice-cold 1x PBS. Collected cells were stored frozen overnight at -80°C in RNAlater (Invitrogen, Cat# AM7023). Frozen cells were thawed at 4°C, washed in 1x PBS, and RNA was isolated using the AllPrep DNA/RNA Mini Kit (Qiagen, Cat# 80204) according to the manufacturer’s instructions. Sequencing libraries were prepared and ran at Fulgent Genetics (Temple City, CA) using NEBNext Ultra II Directional RNA Library Prep Kit (New England Biolabs, Cat# E7760L) and sequenced on Illumina NovaSeq 6000 with S4 flow cell (2 × 150 cycles) with 20 M PE reads per sample.

### Cell lines RNA-seq data preprocessing

Raw sequencing read files (fastq) were pre-processed using the Bioinformatics ExperT System (BETSY) (Chen and Chang, 2017). Sequencing quality was assessed using FastQC and adapter trimming was performed using trimmomatic (0.33) (Bolger et al., 2014). Sequences were aligned using STAR (2.7.6a) (Dobin et al., 2013), followed by counts estimation using HTSeq (Anders et al., 2015) and estimation of gene expression levels using RSEM (1.3.1) (Li and Dewey, 2011). Transcript per million (TPM) values from RSEM were log2(x+1) transformed and filtered to remove genes with the lowest variance (25th percentile) and lowest expression (30th percentile).

### Developing signature for *in vitro* everolimus response using bayesian binary regression

Filtered TPM matrix for the nine cell lines was used to train a supervised Bayesian binary regression model based on the method developed by West et al. (West et al., 2001) and implemented in the GenePattern module SIGNATURE (Chang et al., 2011). Expression values were quantile normalized and a set of 100 features (genes) were obtained that were correlated with the treatment status (DMSO vs. everolimus plus exemestane). A prediction model based on Bayesian regression that used the two metagenes (principal component of the signature gene matrix) with Monte-Carlo simulations was used to obtain classification accuracy in leave-one-out cross-validation (LOOCV) analysis. Based on the successful classification of the cell lines using this model, the selected features were used for further analysis.

### Patient microarray data preprocessing

Gene expression data from patients in a neoadjuvant everolimus trial (Sabine et al., 2010) were obtained from NCBI GEO accession GSE119262. We used expression data from the pre-treatment tumors to train and validated the model. The tumor samples were profiled using Illumina HumanRef-8 v2 Expression BeadChips and quantile normalized using BeadArray (Sabine et al., 2010). We aggregated the expression matrix by first averaging data from multiple probes at the gene level and then averaging the expression levels of replicates. The log-transformed expression levels were standardized such that each gene had a mean = 0 and standard deviation = 1 across the samples.

### Integrative machine-learning framework for response prediction

We implemented a LOOCV framework using the caret package for R (Kuhn, 2021) to combine the *in vitro* signature genes with genes selected from the clinical dataset to develop an integrative biomarker. In each iteration of the cross-validation, we first selected a set of relevant features using Fast Correlation Based Filter for Feature Selection implemented (FCBF) using the FCBF package for R (Lubiana and Nakaya, 2021). We then obtained an integrative signature by combining the *in vitro* signature and the FCBF selected features and used this set of genes as predictors in a random forest model, with the patient response as the outcome variable. This was performed using the randomForest R package (Liaw and Wiener, 2002). An internal cross-validation was performed within each iteration to tune the mtry hyperparameter. All analyses were performed in R version 4.1.0 (R Core Team, 2021).

### Functional enrichment analyses

Pathway enrichment analyses were performed using the g:Profiler2 package for R (Kolberg et al., 2020). Genes in the *in vitro* signature were split into two lists (up or down in everolimus treated cells) and analyzed for enrichment of GO:BP, KEGG, and REACTOME pathway terms. Functional enrichment was performed for the over-representation of genes using the hypergeometric test and adjusted for multiple comparisons. Enrichment plots and tables were created using the g:Profiler2 package, with color coding in the tables showing the level of evidence associated with the terms. A dark blue color indicated weaker evidence whereas an orange color indicated strong, experimentally derived evidence for the term.

## Results

### An integrative machine learning framework

**FIGURE 1:**
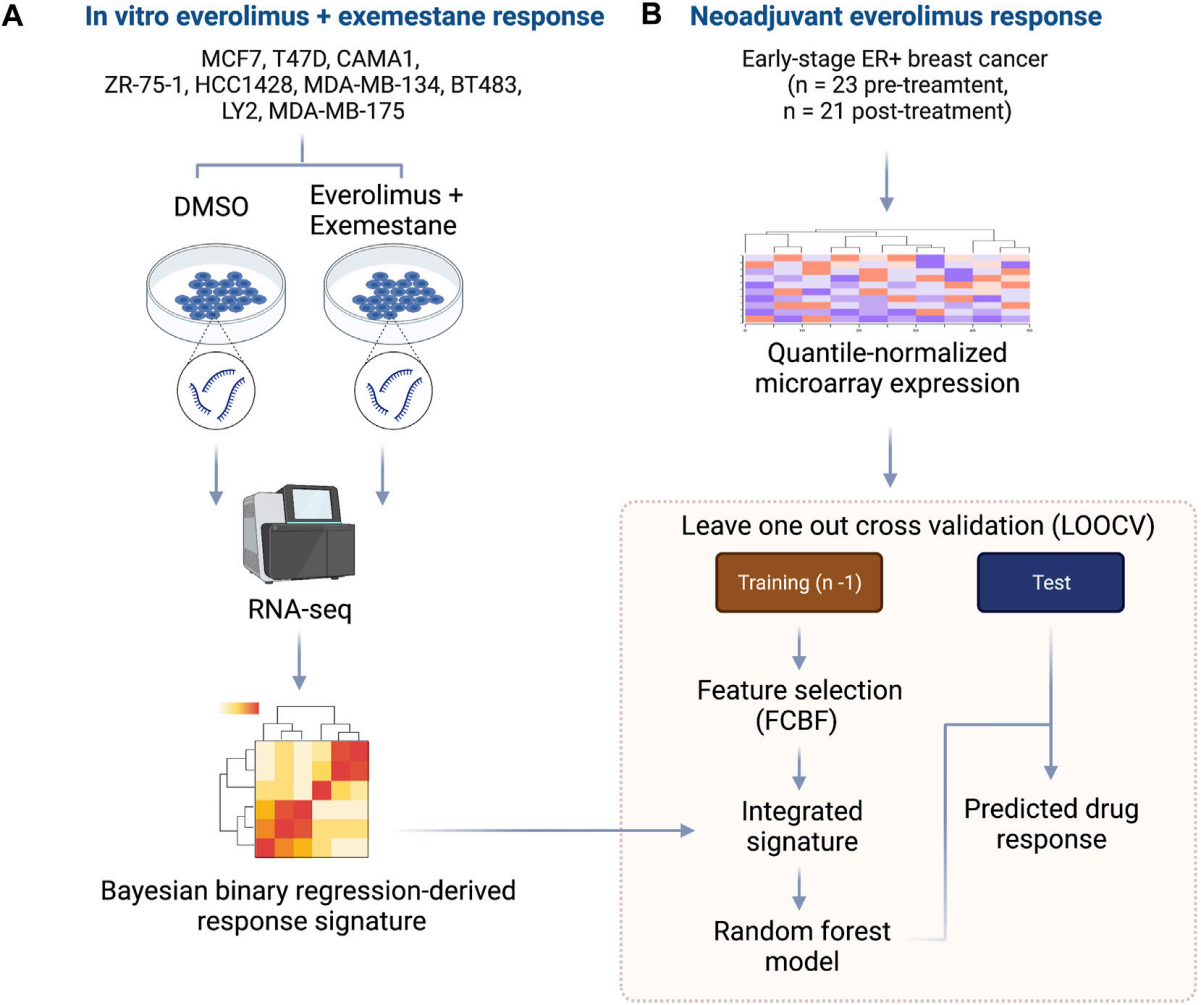
Outline of integrative approach for mTOR inhibitor biomarker development. **(A)** The *in vitro* signature was developed using 9 ER+ breast cancer cell lines. Each cell line was treated with either DMSO or everolimus plus exemestane. Total RNA was extracted, and cDNA libraries were prepared for RNA-seq. The raw transcripts were pre-processed, followed by quantile normalization and feature selection using the Bayesian binary regression framework. **(B)** Microarray data from early-stage ER+ breast cancer patients treated with neoadjuvant everolimus were pre-processed and analyzed within a leave-one-out cross-validation (LOOCV) framework. Each iteration of the LOOCV generated a list of features correlated with response. These features were integrated with the response signature derived from cell lines to obtain an integrated signature. The integrated signature was then used as a set of predictors in random forest model to predict the response in the test sample.

We developed an integrated biomarker development approach that harnessed evidence from controlled *in vitro* experiments with ER+/HER2- breast cancer cell lines treated with everolimus and combined this with data from a neoadjuvant clinical trial of ER+ breast cancer patients treated with everolimus. The outline for our approach is shown in Figure 1. First, we cultured nine cancer cell lines, including MCF7, T47D, CAMA1, ZR-75-1, HCC1428, MDA-MB-134, BT483, LY2 and MDA-MB-175 in either 0.2% DMSO (control) or a combination of 0.5 nM everolimus + 25 µM exemestane. The treatment concentration for the experiment was determined based on the dose-response curves of the nine cell lines (Supplementary Figure S1; Supplementary Table S1). After 6 h of treatment, total RNA was extracted from each pair of untreated and treated cell lines and sequenced at a target read depth of 20 M reads. We then filtered the pre-processed gene expression (RSEM) from each cell line to retain the most informative genes by removing low expression and low variance genes. The expression levels were quantile normalized, followed by feature selection, and fitting a Bayesian binary regression model with treatment status as the outcome (Figure 1A). Concurrently, we obtained gene expression data from pre-treatment biopsies of 23 ER+ breast cancers that received neoadjuvant everolimus for about 2 weeks (Sabine et al., 2010). This trial reported clinical response as a change in Ki67 staining percentage at the end of 11–14 days of treatment, with patients showing more than a 10% decrease in Ki67 staining classified as responders. We then implemented a LOOCV framework that used two sets of features: 1. A set of signature genes from used in the Bayesian binary regression model of the cell lines treated with everolimus and exemestane and 2. A set of features that were selected using FCBF. This integrated set of features was used to train a random forest classifier within each fold of the LOOCV (Figure 1B).

### Transcriptomic signature of *in vitro* everolimus response

**FIGURE 2:**
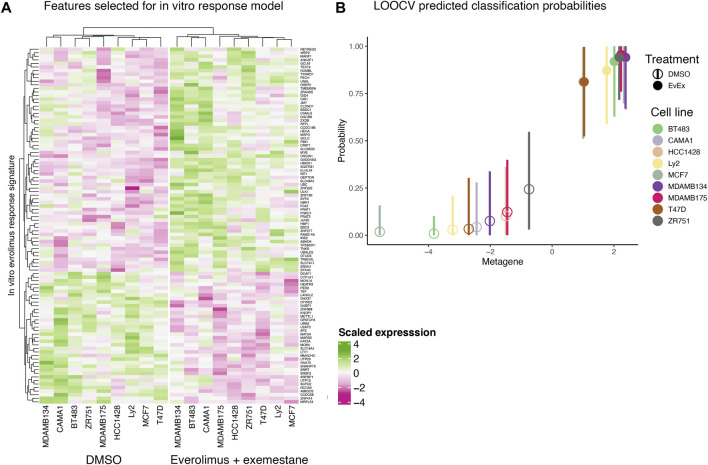
In vitro everolimus response signature and validation. **(A)** Heatmap of genes selected by the binary regression model to classify and predict everolimus response in 9 ER+/HER2- breast cancer cell lines. The genes in the signature are listed in rows and the columns indicate cell lines. Both rows and columns are shown as hierarchical clusters, with the columns split into two clades, resulting in clustering by treatment status (DMSO and everolimus plus exemestane). **(B)** LOOCV analysis of the 9 breast cancer cell lines. Hollow circles indicate cell lines treated with DMSO while the solid circles indicate cell lines treated with everolimus plus exemestane. The X-axis indicates metagene score, calculated from the principal component of the genes in the signature. Y-axis indicates predicted probability of response, with a value closer to 1 indicating response. The vertical bars indicate 95% confidence interval of the prediction probability.

We created an *in vitro* everolimus response signature using RNA-seq profile of the nine ER+ breast cancer cells, with the treatment status (DMSO vs. everolimus plus exemestane) as the outcome variable. Starting with a matrix of filtered gene expression data across cell lines, we first defined the signature set by selecting genes using Pearson correlation that best differentiated the cell lines based on treatment status (Figure 2A). A Bayesian binary regression model was then fit on the first two principal components of the signature gene expression matrix to classify the cells. This model was sampled using a Markov chain Monte Carlo algorithm to obtain posterior probabilities and 95% confidence intervals (Figure 2B). A probability closer to 1 indicated that the signature genes were active in cells treated with everolimus. As shown in Figure 2B, the signature could clearly distinguish cell lines based on treatment status.

**FIGURE 3:**
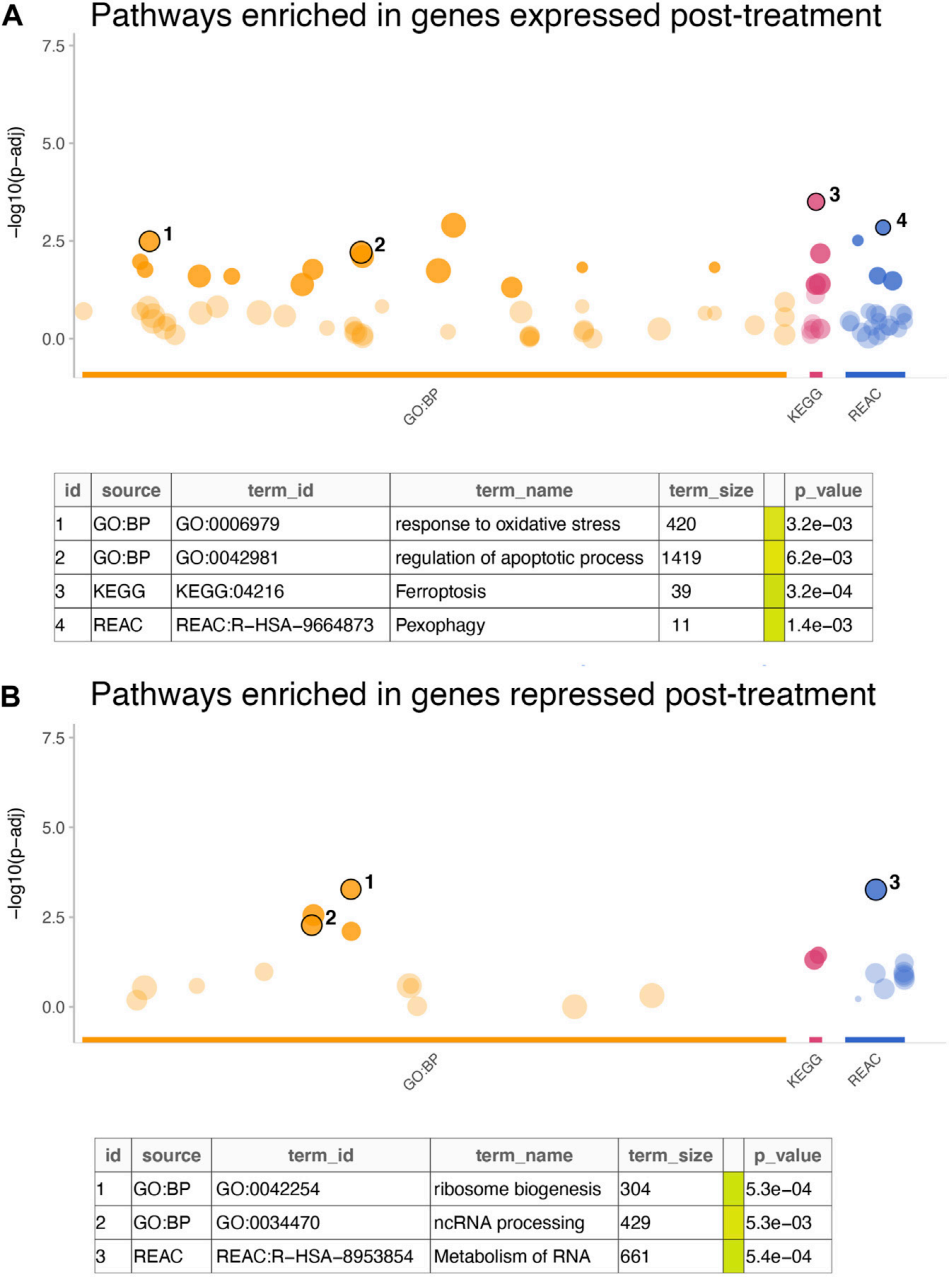
Enrichment analysis of in vitro gene signature. **(A,B)** The dotplots show significance of the enrichment terms from GO:BP, KEGG and REACTOME signatures, with Y-axis showing −log10 of the FDR-adjusted *p*-value from the enrichment test. Key significant terms enriched in genes that were **(A)** expressed at higher levels in the treated cells or **(B)** expressed at higher cells in the untreated cells are annotated in the table below. The term size indicated number of genes in the original signature, while the color code indicates strength of evidence associated with the term. Terms supported by experimental evidence are shown in orange.

Further examination of the signature genes revealed key biological processes and pathways activated or inactivated in the cell lines post treatment. Enrichment analysis for GO:BP, KEGG and REACTOME terms in the genes expressed at higher levels in cells treated with everolimus revealed activation of pathways related to cell death and apoptosis (Figure 3A; Supplementary Table S2). For example, some of the key enriched pathways included response to oxidative stress, regulation of apoptosis, ferroptosis and pexophagy, which are well-known consequences of mTOR inhibition *in vitro*. On the other hand, genes that were downregulated were enriched in terms associated with translation and cell proliferation (Figure 3B; Supplementary Table S3). Again, this agreed with the expectation that mTOR inhibition would lead to reduced protein turnover and proliferation rates.

### Predicting clinical response using integrative model

**FIGURE 4:**
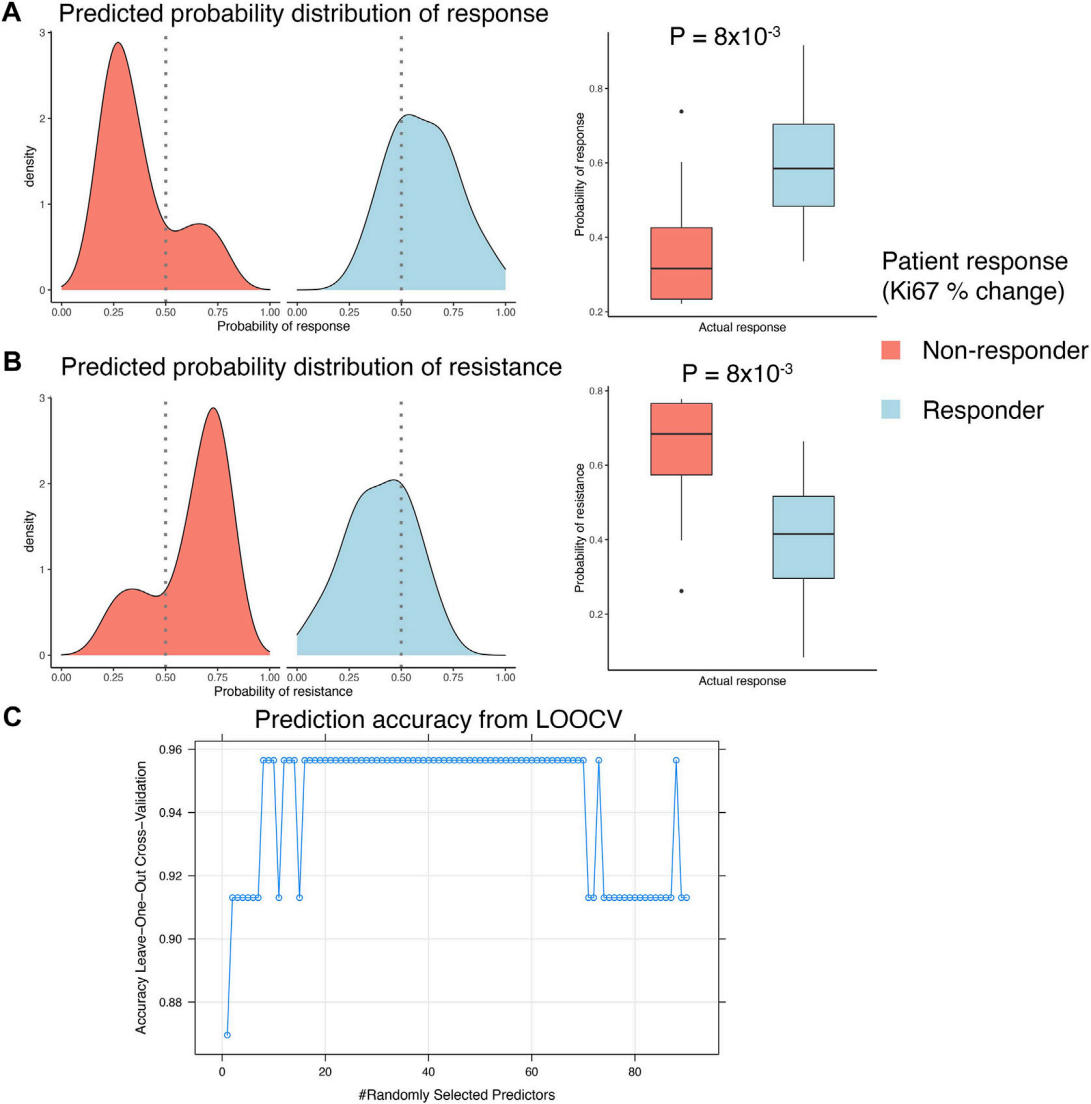
Developing integrated model with patient response data. **(A,B)** LOOCV analysis of the GSE119262 datasets comparing the prediction probabilities of **(A)** response or **(B)** non-response (resistance) calculated using the integrated model combining the *in vitro* response signature with the FCBF-selected features. The density plots on the left show distribution of the prediction probabilities in samples grouped by actual clinical response, with red indicating patients that were clinical non-responders (<10% decrease in Ki67% staining after 2–4 weeks) and blue indicating patients that were clinical responders (>10% decrease in Ki67% staining after 2–4 weeks). The boxplots on the right show statistical comparison of the prediction probabilities between patients grouped by actual clinical response. **(C)** Dot plot showing trends in change of accuracy of the random forest model in LOOCV analysis with varying values of the mtry hyperparameter. An accuracy of ∼0.95 was achieved with mtry = 8 and used to construct the final prediction model.

A clinical study of ER+ breast cancers evaluated everolimus response by measuring percentage Ki67 staining change over the course of treatment of 2 weeks (Sabine et al., 2010). This clinical trial reported response data from 23 pre-treatment biopsies and 21 post-treatment biopsies. We used the pre-treatment gene expression data to develop a biomarker that can predict response to everolimus treatment. This analysis was performed within an LOOCV framework, where each iteration of the cross validation involved selecting relevant features associated with treatment response in the training split, integrating the selected features with the *in vitro* gene signature, and training and validation of a random forest model. We used FCBF algorithm to select the features associated with treatment response. This algorithm utilized symmetrical uncertainty, an information theory derived concept that selected genes with high correlation with the outcome but low correlation with other variables. The genes selected with FCBF were integrated with the *in vitro* signature to train and evaluate the random forest model. LOOCV analysis showed that the predicted probabilities of response based on pre-treatment gene expression data agreed with the actual clinical outcomes, as reported by the clinical trial (Figure 4A). Similarly, the predicted probabilities of non-response agreed with the reported clinical response (Figure 4B). Overall, the random forest model fit on the complete clinical dataset of 23 pre-treatment tumor samples was highly accurate, with consistently high accuracies (>0.9) achieved in the LOOCV analyses for tuning the hyperparameters of the random forest model fit on the full dataset (Figure 4C).

### Identifying potential candidates for everolimus treatment

**FIGURE 5:**
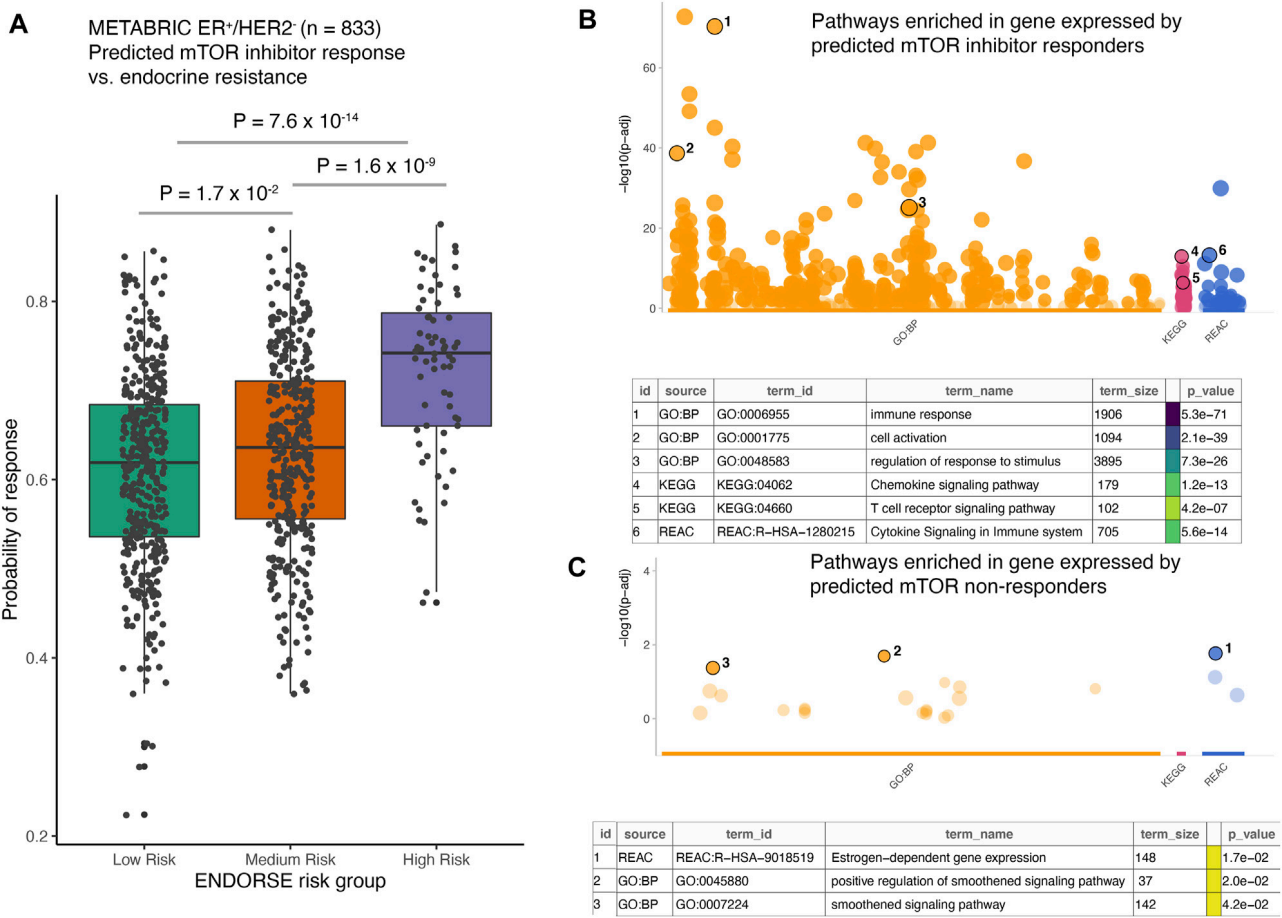
Application of the mTOR inhibitor model on external dataset. **(A)** The boxplots show predicted probability of mTOR inhibitor response in METABRIC ER+/HER2- patients (*n* = 833). The patients were classified as endocrine sensitive (low-risk), endocrine intermediate (medium-risk) and endocrine resistant (high-risk) using the ENDORSE model. The adjusted *p*-values annotated above the boxplots show pairwise comparisons obtained from Tukey’s HSD test applied to a one-way ANOVA model **(B,C)**. The dotplots show significance of the enrichment terms from GO:BP, KEGG and REACTOME signatures, with Y-axis showing -log10 of the FDR-adjusted *p*-value from the enrichment test. Key significant terms enriched in genes that were **(B)** expressed at higher levels in the tumors predicted to be responsive to mTOR inhibitor treatment or **(C)** expressed at higher levels in the tumors non-responsive to mTOR inhibitor treatment are annotated in the table below. The term size indicated number of genes in the original signature, while the color code indicates strength of evidence associated with the term. Terms supported by weak evidence are shown in blue while experimentally derived signatures are shown in orange.

Given the high prediction accuracy of the model in LOOCV analyses, we applied the random forest model trained on the full clinical dataset of 23 pre-treatment samples to predict mTOR inhibitor response in the METABRIC cohort of ER+/HER2- tumors (Curtis et al., 2012). This cohort included 833 breast cancer patients that had received endocrine therapy and were either alive at study completion or died due to the disease. We had previously developed a biomarker model to predict patients with high risk of death on endocrine therapy in this cohort. This model, called ENDORSE, could successfully stratify patients based on predicted endocrine resistance. We had also noted that the patients with ENDORSE risks showed activation of the mTOR signaling pathway. Therefore, we compared the predicted probabilities of mTOR inhibitor response with the ENDORSE classes in the METABRIC cohort. Here, we found that the predicted mTOR response were significantly higher in medium and high-risk groups than the low-risk groups (Figure 5A). Moreover, a large proportion of the high-risk tumors (>40%) showed a high probability of mTOR inhibitor response (>0.75) compared to medium-risk (15%) or low-risk tumors (10%). We further investigated the biological signals enriched in the tumors with a high probability of mTOR inhibitor response (>0.75). Interestingly, we found an overwhelming majority of the biological processes and signaling pathways at the top of the list of significant terms to be associated with immune signaling and communication (Figure 5B; Supplementary Table S4). In contrast, the signatures enriched in non-responders were associated with estrogen signaling or smoothened signaling pathways (Figure 5C; Supplementary Table S5). These suggested the tumors predicted to be non-responsive to mTOR inhibitors were still largely dependent on estrogen signaling or bypassed mTOR signaling *via* the smoothened pathway for growth and proliferation.

## Discussion

The use of mTOR inhibitors such as everolimus has shown promising results in improving outcomes of ER+/HER2- breast cancer patients(Ellard et al., 2009; Baselga et al., 2012; Andre et al., 2014; Piccart et al., 2014). However, there are several treatment options available for progressive and advanced ER+ breast cancers, which include drugs that target the PI3K and mTOR signaling pathways. While the presence of activating mutations on the *PIK3CA* gene guide the use of PI3K inhibitors such as alpelisib (Narayan et al., 2021), the decision to use mTOR inhibitors like everolimus is completely based on patient and provider choice. Therefore, our goal was to systematically develop a new biomarker that may be useful in predicting clinical outcomes for mTOR inhibitors. We have previously developed a prognostic model for endocrine resistance in breast cancer patients using the tumor baseline transcriptomic data (Nath et al., 2022). Here, we extended our approaches to develop a novel machine learning biomarker for everolimus response.

To date, only one clinical trial with any mTOR inhibitor has reported genomic information from breast cancer patients before and after treatment (Sabine et al., 2010). This trial was of limited size of only 23 pre-treatment samples, which made it challenging to train and develop an effective biomarker model. We addressed this issue by first identifying which genes are expressed in response to everolimus in a well-controlled *in vitro* environment. Such approaches have been extensively used to generate gene expression signatures by directly manipulating the expression of genes or using chemical perturbations *in vitro*. For example, the curated and oncogenic signature collection in the molecular signatures database contains over 3000 such signatures generated using genetic or chemical perturbations (Liberzon et al., 2011) that are frequently used in prognostic and drug response signatures (Sonachalam et al., 2012; Tan et al., 2019; Kong et al., 2020; Zeng et al., 2022).

As the *in vitro* environment is less affected by the inter-sample variances typically observed in animal and patient-derived data, our approach allowed us to pick genes with high confidence that show a significant change upon everolimus treatment and are likely good candidate features for a machine learning biomarker model (Figure 2). These genes were sensible and associated with expected biological phenomenon (Figure 3). We then implemented an approach that leveraged pre-treatment tumor transcriptomes and clinical outcomes data from the 23 patients combined with the *in vitro* signature. This integrated model was highly accurate in predicting clinical everolimus response in the LOOCV analyses of the patient data (Figure 4).

We further applied the biomarker to predict mTOR inhibitor response in an independent cohort of ER+/HER2- breast cancer patients from the METABRIC study (Curtis et al., 2012). We obtained gene expression and overall survival data from 833 patients. These patients had received only endocrine therapy and were classified using a prognostic model that predicted risk of death on endocrine therapy (Nath et al., 2022). We had previously found that the METABRIC patients with high risk of death on endocrine therapy showed elevated pathway activity of PI3K/AKT/mTOR signaling pathway (Nath et al., 2022). By applying our biomarker, we found that indeed a vast proportion of the predicted mTOR responsive patients were those in the high risk group (Figure 5). Activation of mTOR signaling is a well-documented phenomenon associated with endocrine resistance and poor prognosis of ER+ breast cancer patients (Ciruelos Gil, 2014; Paplomata and O'Regan, 2014; Dong et al., 2021; Nunnery and Mayer, 2020). Thus, our novel biomarker could be useful in identifying the patients that are most likely to benefit from mTOR inhibitor treatment.

Another interesting aspect of our study were the biological signatures and pathways activated *in vitro* upon everolimus treatment and the ones enriched in patients predicted to be responsive. The *in vitro* signature largely showed enrichment of expected biological pathways, including cellular oxidative stress (Piao et al., 2014), autophagy (Crazzolara et al., 2009) and apoptosis (Tai et al., 2017). In comparison, the patient data showed a large proportion of immune activation pathways as the most significant signatures. mTOR signaling is well-known to play an important role in directing adaptive immune response by receiving microenviromental signals and activating T-cells and dendritic cells (Delgoffe and Powell, 2009). In the tumor microenvironment, mTOR signaling regulates the activity of macrophages and T-cells through inflammatory factors like IL-10, TGF-beta, and membrane bound CTLA-4 and PD-1 (Kim et al., 2017). This has been linked with a shift in balance from an anti-tumor to a pro-tumor immune microenvironment by reducing the proportion of anti-tumor CD8^+^ T-cells and increasing the proportion of Treg and tumor-associated M2 macrophages (Kim et al., 2017; Mafi et al., 2021). Thus, the enrichment of these immune activation-related terms in the patient data captures a known effect of elevated mTOR pathway activity and supports the biomarker-driven classification of the patients as likely responders to mTOR inhibition. We also observed an enrichment of smoothened receptor pathway signatures in the mTOR-resistant tumor. As a major component of the hedgehog signaling pathway, both the canonical and non-canonical activation of the smoothened pathway has been linked with stem-cell like traits, invasiveness and metastatic progression of breast cancers (Jeng et al., 2020). Consequently, multiple interventions targeting the hedgehog and smoothened signaling pathway are currently being evaluated in breast cancer (Bhateja et al., 2019).

Key limitations of this study are lack of a large-scale training dataset and an additional independent validation dataset for the biomarker model. The clinical training data used in the study consisted of only 23 pre-treatment samples, with a large number of potential predictive features. We attempted to mitigate this challenge by systematically reducing the number of predictive features using the *in vitro* signature and selecting a limited number of empirical features from the clinical datasets for model construction. Furthermore, we performed the model development and validation in a LOOCV framework, where the empirical features from the clinical dataset were only picked from the training split. The model was then applied to predict outcome in the left-out test sample. This approach helped in diminishing problems associated with overfitting models to the data and overestimating model accuracies. Nevertheless, clinical translation of the biomarker will benefit greatly from additional validation and refinement using prospective biopsies or through retrospective analyses of banked samples.

Given that patients progressing on endocrine therapy have multiple treatment options, including aromatase inhibitors, chemotherapy, PI3K inhibition or mTOR blockage, development of biomarkers to guide therapy selection of these patients can help ensure they are treated with the most effective drug regimen. This study uses both experimental and patient-based data to develop a biomarker for response to everolimus, and to understand the signaling underlying inhibition of mTOR signaling in ER+ breast cancer.

## Data Availability

The neoadjuvant everolimus clinical trial dataset used in this study can be found in the NCBI GEO repository under the accession GSE119262 (https://www.ncbi.nlm.nih.gov/geo/query/acc.cgi?acc=GSE119262). The METABRIC dataset can be obtained from the cBioPortal for cancer genomics (https://www.cbioportal.org/study/summary?id=brca_metabric). Scripts associated with this manuscript are available at https://github.com/aritronath/Everolimus_MLB. The GenePattern BinReg module, results, and the RNA-seq input data for the nine ER+ breast cancer cell lines analyzed in this study are also available in this GitHub repository under “releases”. Genes in the final integrative signature are provided in Supplementary Table 6.
