# Supplementary figures and images for: Predicting clinical response to everolimus in ER+ breast cancers using machine-learning
Source: Front Mol Biosci. 2022 Oct 11;9:981962. doi: 10.3389/fmolb.2022.981962 (PMC9592823; doi:10.3389/fmolb.2022.981962)

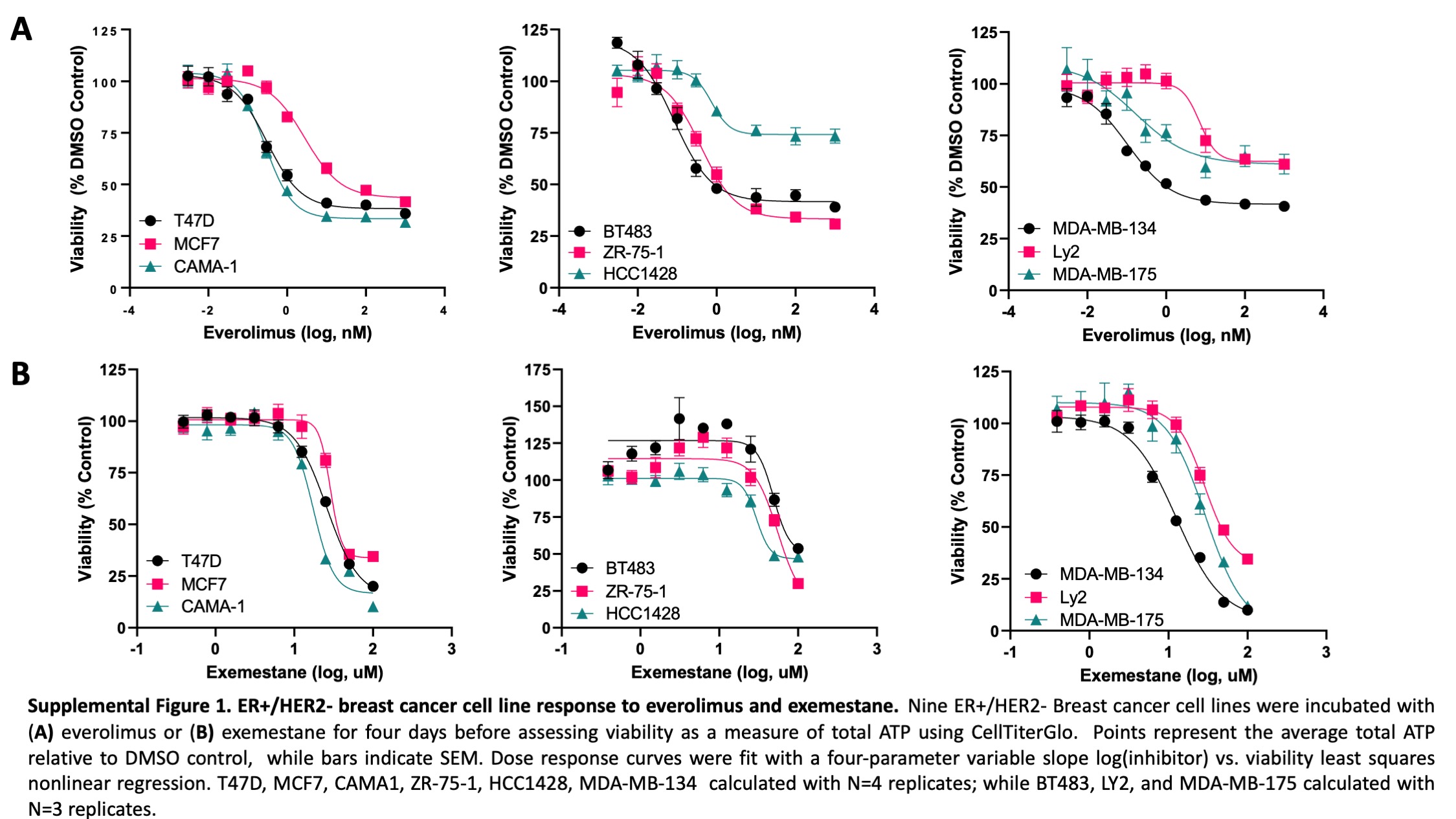

Supplement: Supplementary file 3 [file Image1.JPEG]
